# The Penetration–Explosion Effects of Differently Distributed Inactive/Active Composite Shaped Charge Jets

**DOI:** 10.3390/ma15010344

**Published:** 2022-01-04

**Authors:** Jiacheng Peng, Jianwei Jiang, Jianbing Men, Jinlin Li, Dongkang Zhou, Yuan Hu

**Affiliations:** 1 State Key Laboratory of Explosion Science and Technology, Beijing Institute of Technology, Beijing 100081, China; pjc13701252487@163.com (J.P.); menjb@bit.edu.cn (J.M.); lijinlin219@163.com (J.L.); 2 Anhui Fangyuan Mechanical & Electrical Co., Ltd., Bengbu 233000, China; 18605529104@163.com (D.Z.); huyuan15675528565@163.com (Y.H.)

**Keywords:** inactive/active double-layer liner, composite jets, penetration–explosion effects, shaped charge

## Abstract

An analysis of the penetration–explosion (PE) effects of four distributions of inactive/active composite jets shows that a well-designed inactive/active double-layer liner can promote composite jet damage. Penetration experiments were then carried out for shaped charge jets having a single inactive (Cu) liner or an inactive/active (Cu/Al) double-layer liner with variable liner height. The behaviors and firelight patterns of the different jets were captured by high-speed photography. The perforation, deformation area, and deflection were measured for each plate, showing that the Cu/Al jets have stronger PE effects. Numerical simulation shows that the tip of the composite jet generated from the full-height liner is only Cu, whereas for the other jet, from the double-layer liner, Cu is almost wrapped entirely by Al.

## 1. Introduction

Shaped charge jets have remarkable armor-piercing capability and are popular in high explosive anti-tank (HEAT) warheads [1]. With smart guidance widely applied in HEAT, shaped charge jets can avoid the strongest protection and focus on the weakest point of the armor. As a result, depth of penetration is no longer the only merit indicator of shaped charge jets. Instead, severe damage to internal targets becomes the primary objective. Current research aims to develop active jets with both good penetration depth and powerful aftereffects.

Reactive structure materials (RSMs) can be directly processed into active liners, and these will release substantial chemical energy under heating or high-speed impact [2,3,4,5,6,7]. In 2001, Baker et al. reported that the high-speed jet formed by an active liner under explosion can achieve five times the destructive power of using Al alone [8]. Laszlo found that the jet formed by the energetic liner of zirconium-based material creates greater lateral damage [9]. Davison and Pratt introduced an active jet with an energetic sandwich liner that can penetrate concrete with a deeper but smaller hole [10]. Church et al. proved a metallic reaction by recovering the Al/Ni particles of active jets and detecting the intermetallic compound Al/Ni [11]. Zhang et al. designed Al/Ni active jets that can significantly increase the perforation diameter [12]. Guo et al. found that Al/PTFE (polytetrafluoroethylene) active jets can damage steel ingots much more powerfully, even though their penetration depth is much less [13]. The formability of active jets directly made of RSMs is generally poor due to the low density and poor strength of RSMs. These jets have much less penetration depth than traditional Cu jets although they have much stronger lateral effects on the targets [14,15,16,17].

For jets with inactive/active double-layer liners, it is expected that the inactive part of the composite jet will first penetrate the target, and the active part will then generate explosion-like damage inside the hole. Xu et al. took flash X-ray photos of a double-layer liner compacted into a stable, slender, high-speed metal jet, whose tail is mainly composed of the active materials [18]. Lee et al. deposited Al and Al-Ni powders as active coatings on Cu liners by dynamic spraying and carried out comparative penetration experiments to study the PE effects of the composite jets [19,20,21]. Guo et al. verified that the jets composed of PTFE/Al and Cu penetrate more deeply than single active jets [22]. Huang et al. designed a K-shaped charge with a double-layer liner consisting of Al/Ni and Cu, and the composite jets created much deeper penetration on steel and concrete targets [23]. Han et al. constructed a tungsten and zirconium alloy liner and showed in experiments that the composite jets achieved much deeper penetration [24]. Zhang et al. found that compared with jets using Cu liner, the Cu-PTFE/Al double-layer liner jet generated a penetration cavity diameter twice as large and a spalling area four times as large [25]. Wang et al. studied a reactive material double-layered liner shaped charge and investigated its penetration into thick steel targets, and found that the defeat mechanism includes penetration by the precursor Cu jet as well as the energy release by the subsequent PTFE/Al active component [26].

The inactive/active double-layer liner can efficiently damage targets with its PE effects. Because the inactive and active parts have different impacting characteristics, composite jets with various distributions should presumably possess different PE effects. Research is still lacking on the PE effects caused by different double liners with the same inactive/active materials, as well as on the structure matching of the double liners.

In this paper, different PE effects of four distributed jets were analyzed. In the ensuing penetration experiment, multilayer targets were penetrated by an inactive (Cu) jet and two inactive/active (Cu/Al) composite jets. The perforation and deformation area and the deflection of each plate were measured. The characteristics of firelight were captured by high-speed photography, which demonstrates distinct PE effects that can be attributed to the different distribution of composite jets. The same effects were obtained by simulating the jet formation numerically, and the experimental and simulation results were well associated.

## 2. PE Effects of Jets with Different Elemental Distribution

**Figure 1:**
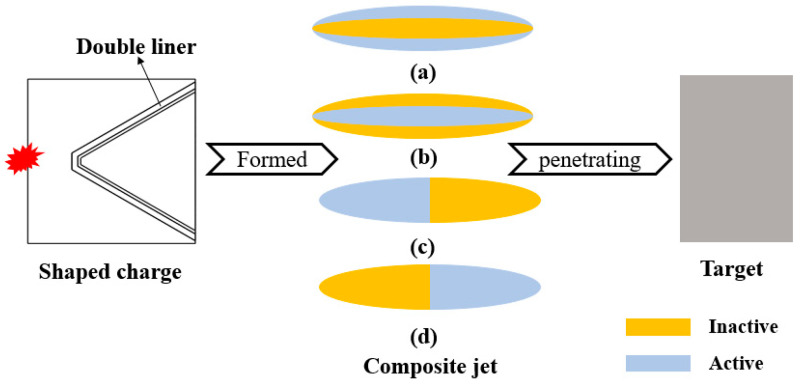
Elemental distribution within composite jets: (**a**) outer Al and inner Cu; (**b**) outer Cu and inner Al; (**c**) front Cu and rear Al; (**d**) front Al and rear Cu.

The composite jets generated from an inactive/active double-layer liner are impermeable and unspaced under extreme loading, are axisymmetric and rotational, and have a defined distribution once formed. Figure 1 shows the four basic distributions that can reflect the properties of the inactive/active composite jet, in which the yellow color represents the inactive part (Cu) and the silver-white color represents the active part (Al, obtained by cold spraying instead of the traditional machining to improve the activity). In the composite jet, Cu has much stronger penetration ability than Al, but Al causes much stronger aftereffects. The explosion-like reaction of Al also needs the high-speed impact that is necessary for activating RSMs [21].

**Figure 2:**
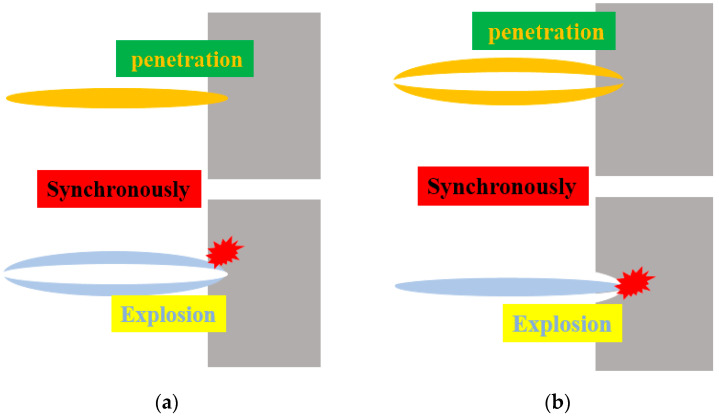

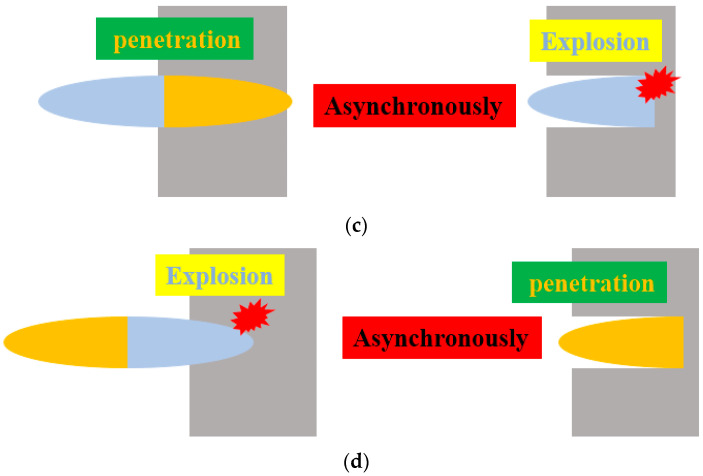
PE effects of different distributed jets: (**a**) Inner Cu and outer Al; (**b**) Outer Cu and inner Al; (**c**) Front Cu and rear Al; (**d**) Front Al and rear Cu.

Figure 2 illustrates the PE effects of jets with the same velocity gradient with different distributions. For distribution (a), the target shows deep perforation and small aperture because the interior Cu jet has excellent penetration power. The tubular Al jet wrapping the Cu jet is impacted and activated synchronously, as it has the same velocity gradient. As a result, there is intense firelight, and the target experiences extensive lateral damage such as expanded perforation and deformation. For distribution (b), the wrapping tubular Cu jet cannot create a deep hole in the target because it is weaker than the Cu jet in distribution (a). The explosion-like effect occurs when interior Al jet impacts the undamaged center, which may interfere with the synchronous penetration of the Cu jet. For distribution (c), the leading forceful Cu jet enables very deep and thorough penetration such that the trailing Al jet easily follows through the perforation without being activated. The PE effect is out of sync due to the front and back distribution of Cu and Al, and the reaction occurs only when the Al jet hits the perforated sidewall or the bottom that is not entirely eroded. For distribution (d), the high-speed Al jet located at the head penetrates the target and is activated to give an explosion-like reaction at the same time, which results in a shallow pit with a large diameter on the target. The trailing Cu jet will deepen the penetration with relatively low velocity after the active Al has been consumed. The overall penetration of this composite jet is the weakest because of the poor distribution, and its PE effect is not synchronized.

## 3. Penetration Experiment

### 3.1. Experimental Setup

**Figure 3:**
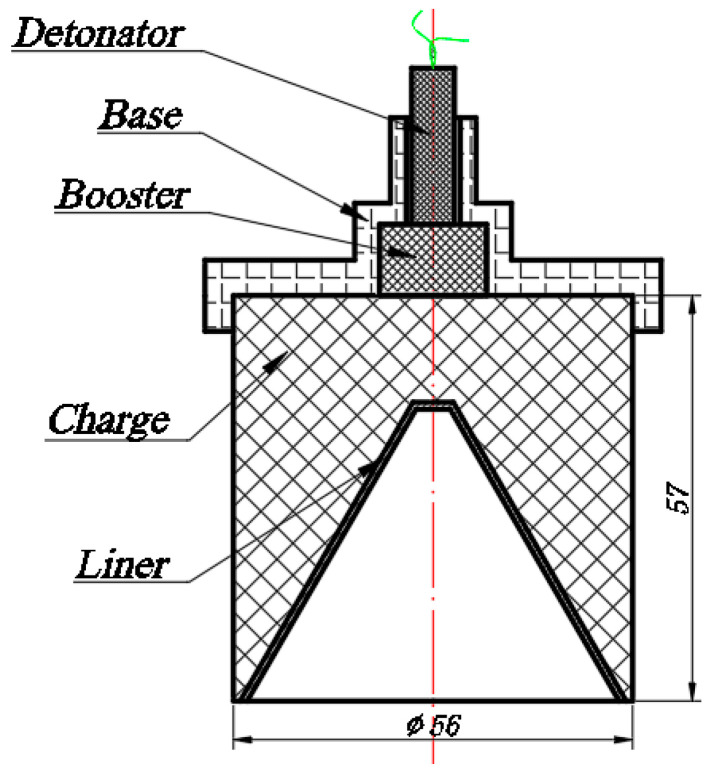
Structure of the 56 mm caliber shaped charge.

Three designs of shaped charge are prepared, all based on the structure of a 56 mm caliber shaped charge shown in Figure 3. The charge in Figure 3 is referred to as Design 1 and used as the control. The liner is 1 mm thick and made from copper. The liner has a cone shape with the top angle being 60° and the tip is truncated by machining. The main charge is RDX-based explosive JH-2 [27] (also known as 8701, ~1.71 g/cm^3^) pressed at room temperature. The booster is JH-14 (96.5% RDX, 3.5% fluorine rubber and colloidal graphite, ~1.70 g/cm^3^) pressed at room temperature. The base of the detonator is made from resin by 3D printing.

**Figure 4:**
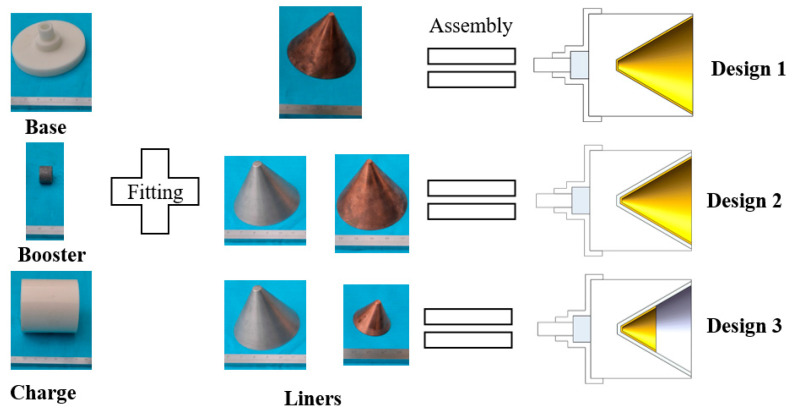
Configuration of the three designs.

Figure 4 shows the details of the other two charges. Design 2 contains a double-layer metal liner. The inner layer is the active liner, which is a truncated cone with a uniform thickness of 2 mm. It is prepared by block machining and Al particles about 15–45 μm in size were deposited on the substrate by cold spraying to improve the activity of the liner material [14]. The inactive outer layer is 1 mm machined Cu. The rest is the same as in Design 1. Design 3 differs from Design 2 in that the outer Cu liner is half the original height. Table 1 lists the detained parameters of the three charges.

**Figure 5:**
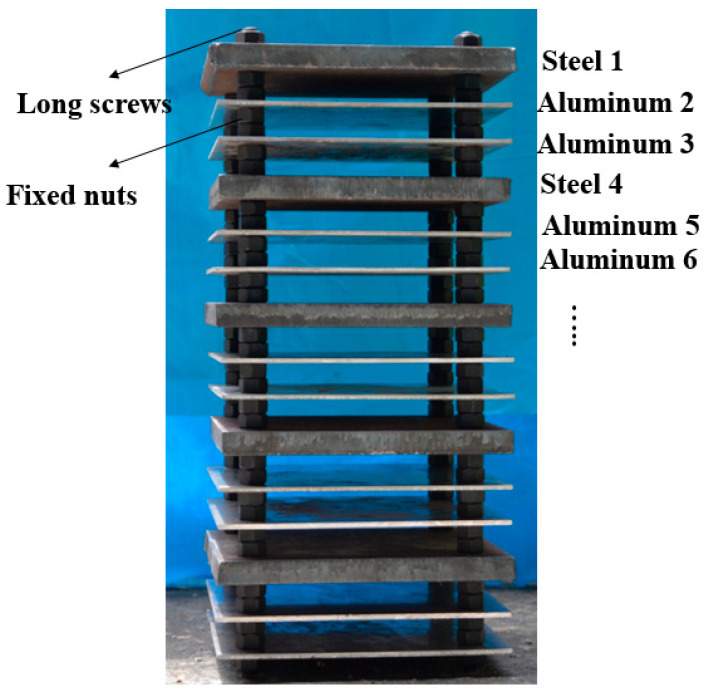
Photo of a marked multilayer target.

Because of the jet’s strong penetration ability, the penetration experiment used multi-layered targets consisting of a series of 160 mm × 160 mm steel-aluminum-aluminum plates [14]. The 15 mm thick 45# steel plate was used to interfere with the jet’s penetration and initiate the active part of the composite jet, and the two 3 mm thick Al-2024 aluminum plates were used to measure the aftereffects. Neighboring plates were spaced 20 mm apart to observe the behavior and the firelight of the impacting jet by high-speed photography. This trio of steel-aluminum-aluminum plates was repeated five times to give 15 plates in total in the target, because the penetration depth and the aftereffect of different composite jets cannot be obtained effectively if all plates are penetrated completely, and each trio allows measurement of the jets’ potential PE effects in a stepwise manner. All plates were fixed with four 12 mm caliber long screws and standard nuts on both sides, as shown in Figure 5. Steel plate 1 in Figure 5 is referred to as S-1, and aluminum plate 2 is referred to as A-2, etc.

**Figure 6:**
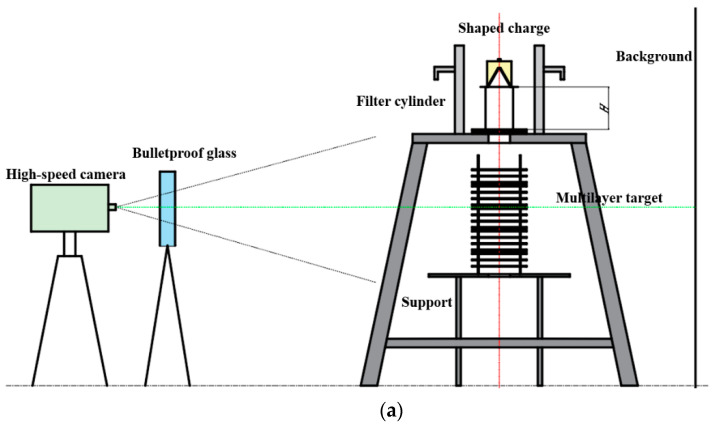

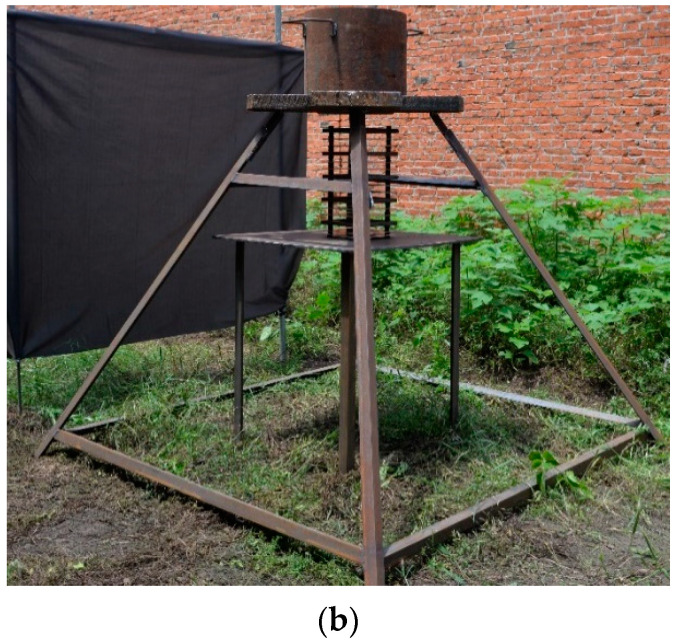
Setup of the penetration experiment: (**a**) schematic; (**b**) photo.

Figure 6 shows the schematic and the experimental setup of the jet penetration experiment. All specimens were placed in a protective steel cylinder with a uniform thickness of 30 mm to prevent lateral explosion. The first steel plate of the multilayer target tower was placed on the surface of a big support to filter the intense light leaking from the channel at the time of the explosion, and other plates were on the small support. The surface levels of the large and small supports were at 1350 mm and 780 mm high from the ground. A dark gray background was set behind the supports to improve the quality of the images. The blasting height was set to 200 mm by placing a PVC cylinder on the first steel plate. The FASTCAM high-speed photography device has a shooting frequency of 100,000 fps. A thick pane of bulletproof glass was placed in front of the camera to protect the equipment.

### 3.2. Destruction of Multilayer Targets

**Figure 7:**
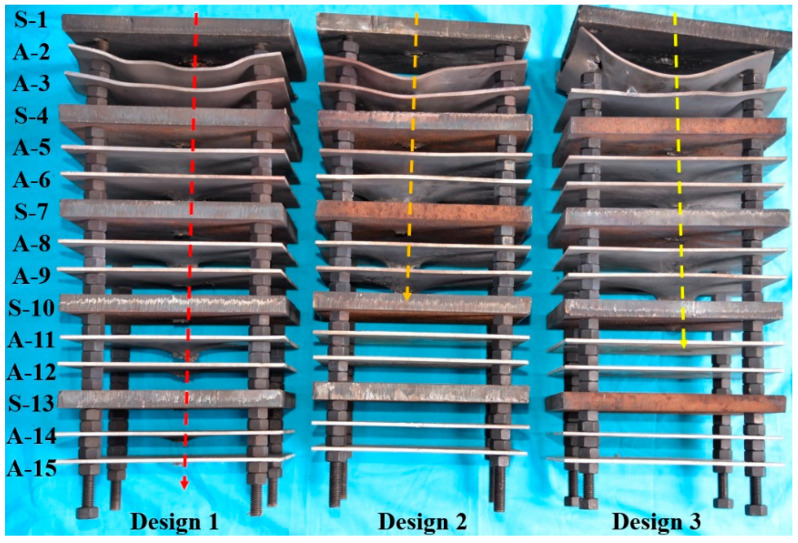
Damaged multilayer targets.

Figure 7 shows the damaged multilayer targets for the different charge designs. We use $P_{N}$ to represent the penetration ability of design *N*. For the single Cu jet (design 1), we have $P_{1}$ > A-15 because all 15 plates were completely penetrated. Similarly, for the composite Cu/Al jet of design 2, A-9 < $P_{2}$ < S-10 because the penetration was limited to only the 9th plate. The composite jet of design 3 penetrated the 10th steel plate but not the 11th Al plate, and thus S-10 < $P_{3}$ < A-11. That is, the penetration ability of the three designs falls in the order of $P_{1}>P_{3}>P_{2}$.

**Figure 8:**
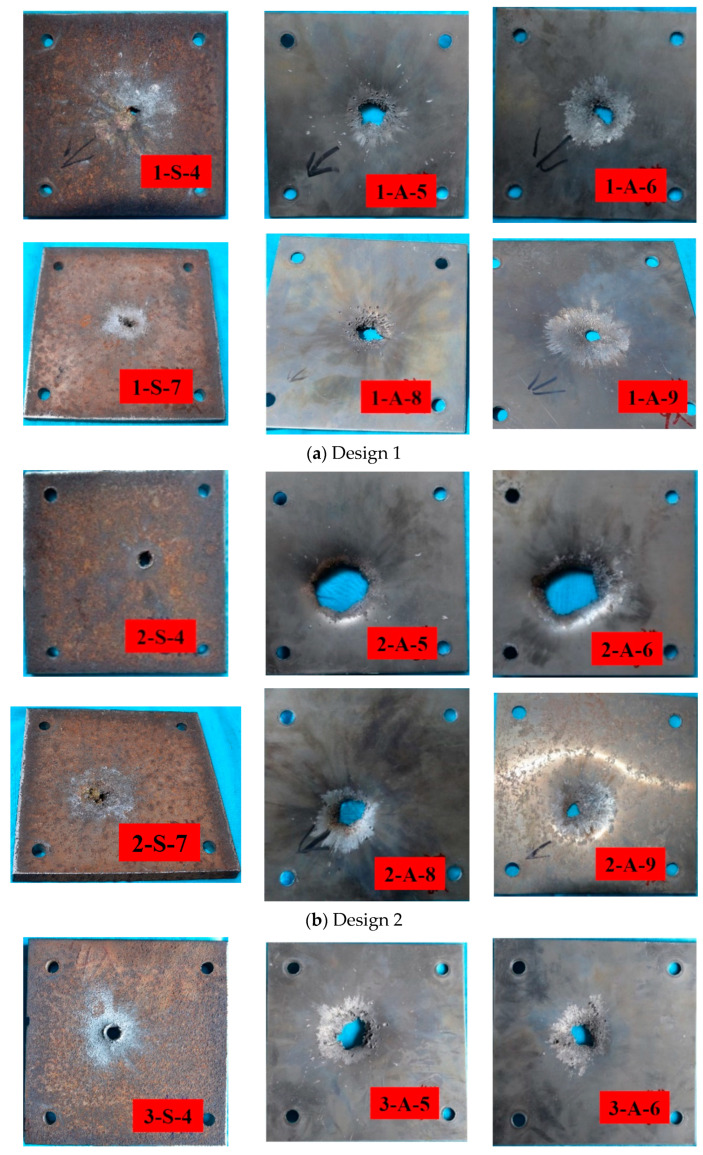

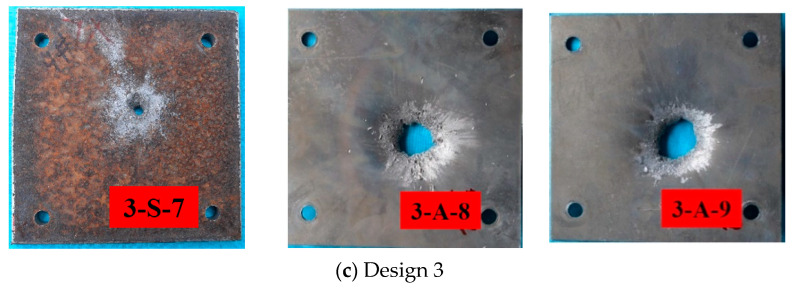
Impacted steel and aluminum plates in penetration experiments for design 1~3 (**a**–**c**): S for the steel plate and A for the aluminum plate.

The strong shockwave caused by detonation at the steel plate is concentrated at the large aperture on the rear of the first steel plate and directed to the two subsequent aluminum plates to realize additional damage (Figure 7). Figure 8 shows the typical plates in the middle of multilayer targets after jet penetration. Perforation, deformation, and deflection were measured for each plate.

**Figure 9:**
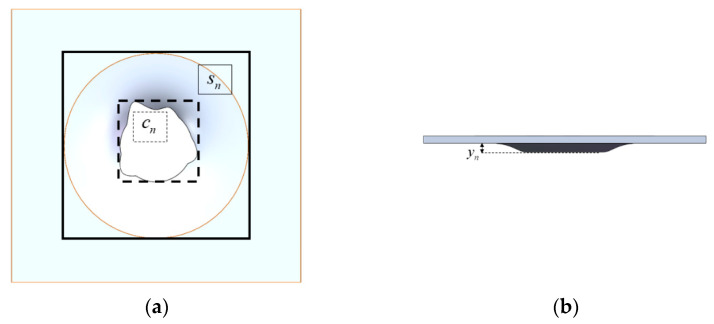
Evaluation of impact on plates: (**a**) top view; (**b**) side view: $c_{n},s_{n}$ and $y_{n}$ for the perforation area, deformation area and deflection of plate *n,* respectively.

**Figure 10:**
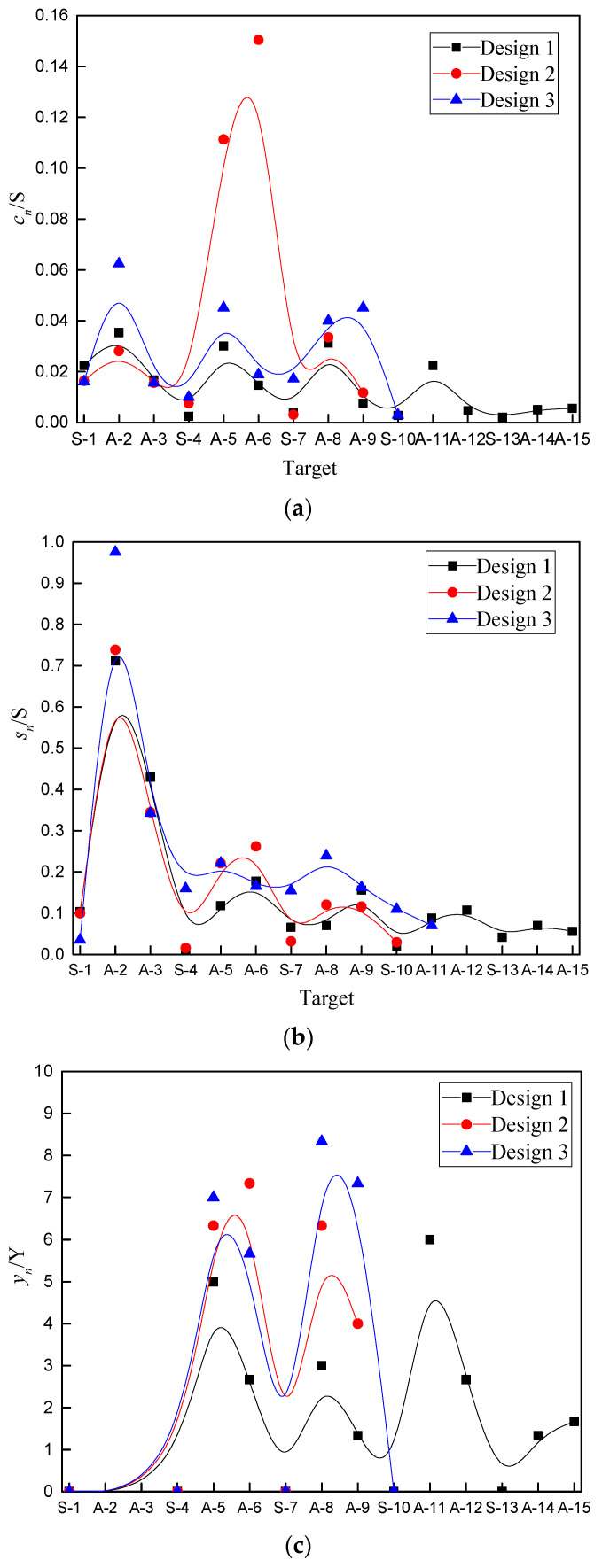

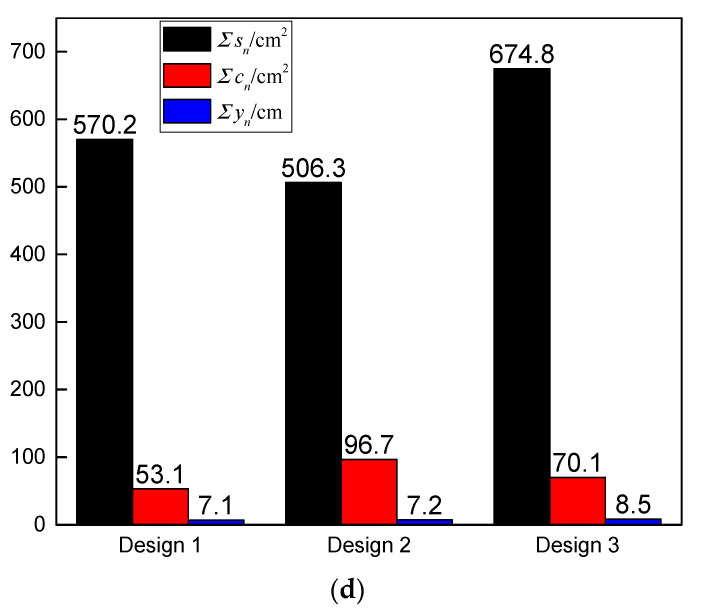
Damage to impacted plates.: (**a**) perforation; (**b**) deformation; (**c**) deflection; (**d**) accumulated damage: S for the steel plate and A for the aluminum plate.

The composite jets create much stronger perforation and deformation on the Al plates than the single Cu jet, and there are obvious PE effects from design 2 and design 3 when the Cu/Al composite jets hit the plates. Figure 9 shows how the impact created by design *n* is evaluated, where $c_{n}$ and $s_{n}$ are the areas of the outer rectangles that cover all perforation and deformation of each plate, and the deflection $y_{n}$ is calculated as the maximum distance from the warping edge of the hole to the lower surface of the plate. Figure 10 gives the ratio of $c_{n}$ and $s_{n}$ to the plate’s original area of *S*, the ratio of $y_{n}$ to the plate’s thickness *Y*, and their accumulation (i.e., $\sum c_{n}$, $\sum s_{n}$, $\sum y_{n}$) for the three designs.

Figure 10d shows that the total deflection is almost the same for all three jets, and only design 3 has a slightly larger cumulative deflection. Note that deflection was not counted in Figure 10 for A-2 and A-3 because it would exceed the plate spacing (20 mm) if it was not blocked by S-4. However, the cumulative perforation and deformation vary significantly. Specifically, total perforation ranks in the order of design 2 > design 3 > design 1, and total deformation ranks in the order of design 3 > design 1 > design 2.

When penetration occurs quickly enough, $c_{n}$, $s_{n}$, and $y_{n}$ will all be small. Thus, PE effects were assessed by comparing with design 1. The maximum perforation is less than 20% of the plate’s original area, which is much smaller than the maximum deformation. The steel plates hardly produce any deflection whereas the Al plates contribute most of the deflection, because the former is much thicker (15 mm) than the latter (3 mm).

The PE effects are stronger on the Al plates once the active part of the composite jets is activated upon impact. The plates A-5 and A-6 in design 2 have much larger perforation than any other plates of all designs. For design 3, the PE effects are spread out more evenly, and perforation and deformation are largely uniform for all penetrated plates. Compared with design 1, design 3 has poorer penetration but significantly stronger accumulated deformation and perforation. In all, with the deformation of plates as the primary consideration, the aftereffect of the three designs can be ranked as $E_{3}>E_{2}>E_{1}$, where $E_{N}$ represents the aftereffect of design *N*.

### 3.3. Impacting Behavior and Firelight

**Figure 11:**
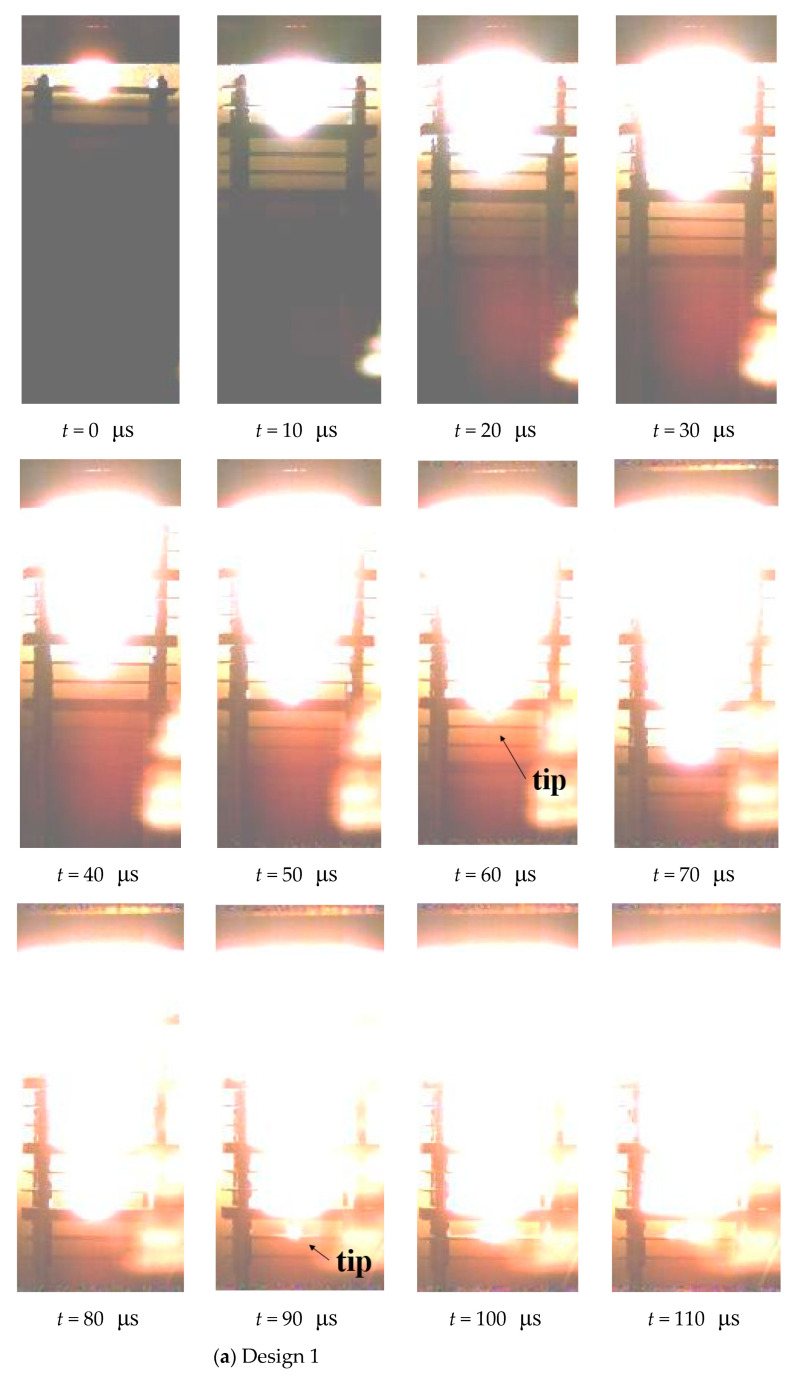

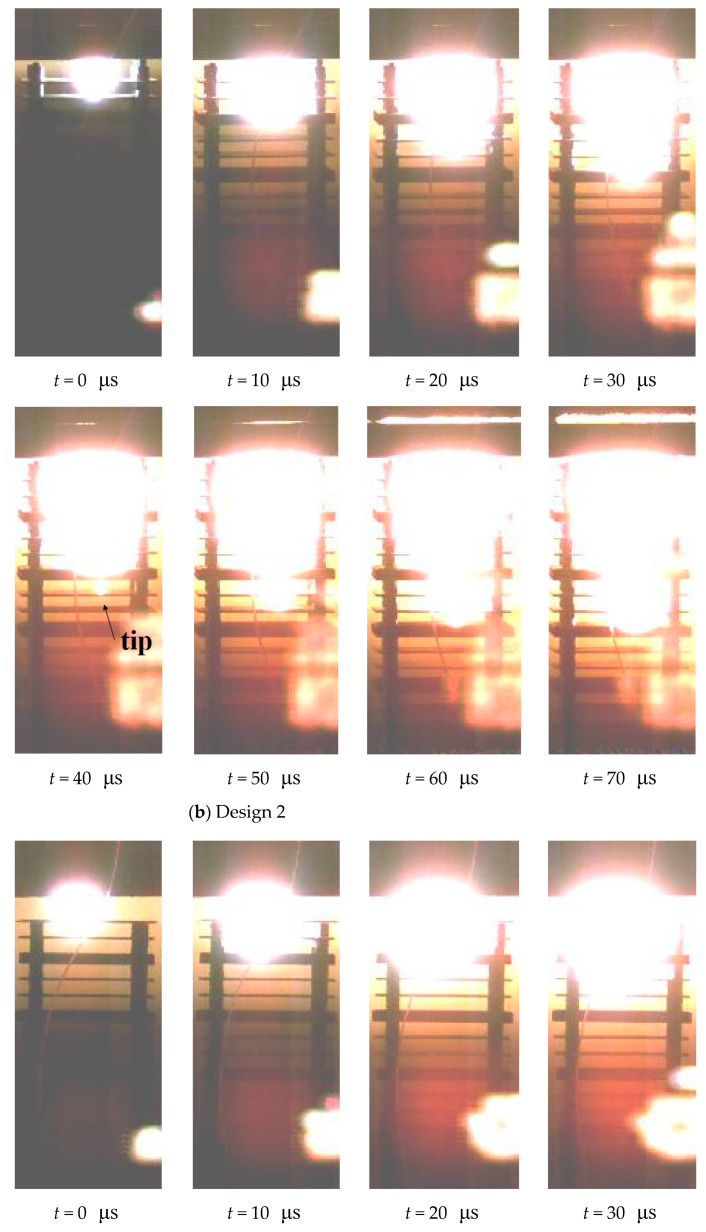

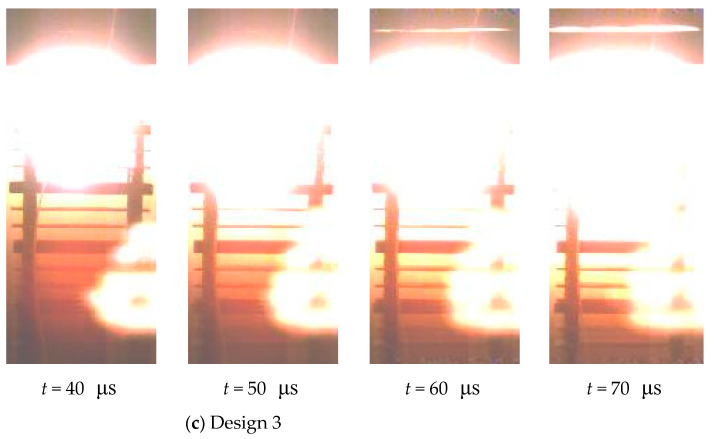
Photos of moving jets and firelight for design 1~3 (**a**–**c**). Note the light that appears at the lower right of each photo is a reflection from the bulletproof glass.

Figure 11 shows the photos and the firelight for the three designs, where *t* = 0 is the instant when A-2 is hit.

Figure 11 shows the jet, the target plates, and the flame from the explosion, and all bright light comes from high temperature or from the impact of high-speed jets on the plates. It is difficult to distinguish the contour of the jet on the image, and the lowest point of the bright color is the jet tip that travels the fastest. The velocity of the jet tip can be calculated by the penetration distance from 0 to 30 μs, including the gap and thickness of the target plates. The jet tip penetrates several more plates after going through the first Al plate, and the average velocity is 4133, 3867, 3467 m/s for design 1, 2, and 3, respectively.

The luminous area of the single Cu jet (design 1) increases gradually from the tip to the tail, and the outline of the firelight is like an inverted isosceles triangle (Figure 11a). The slender and bright jet tip can be seen at *t* = 60 μs and *t* = 90 μs after the thick steel plates are penetrated, and the firelight is mild when the tip hits the steel plates but strong when it hits the Al plates. For design 2 (Figure 11b), intense firelight occurs after the tip has quickly penetrated the plate upon the impact of the subsequent jet. The outline of the firelight has the shape of a cylinder, which changes into two stacked cylinders of different sizes after 40 μs. The bright and slender tip can be seen at *t* = 40 μs and it is thinner than in design 1, but it gradually expands after penetrating the Al plates. For design 3 (Figure 11c), the tip cannot be captured because the composite jet expands rapidly to a large fire when hitting the target. The outline of the firelight has an oval shape. The different PE effects produced by the two composite jets come from the distribution of inactive/active components, which depends on the double-layer liners. The actual distribution will be obtained by the simulation in the following.

## 4. Numerical Simulation of Jet Formation

### 4.1. Numerical Setup

**Figure 12:**
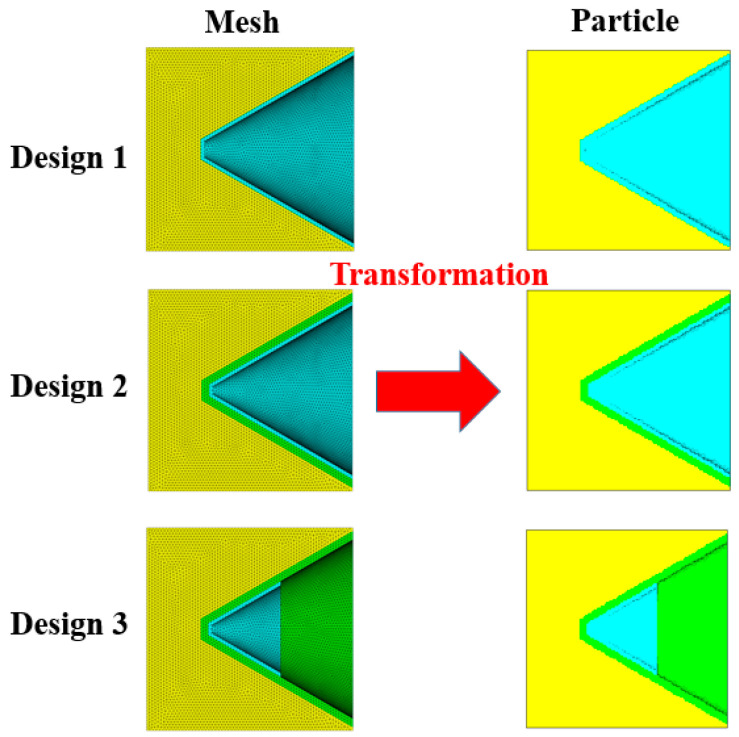
Transformation diagram.

The smooth particle hydrodynamics (SPH) algorithm is employed to account for the collapse and severe deformation of the liners. Tetrahedral grids were established based on the simplified configurations of three designs and then transformed into a uniform SPH particle model, and there are no negative grids. A uniform mesh of 0.3 mm SPH grids is used globally in Autodyn-3D to ensure the calculation accuracy as well as the computational efficiency (Figure 12).

The liner materials (Cu and Al) adopt the Johnson-Cook model with shock EOS. The Johnson-Cook model represents the strength behavior of typical metals subjected to large strain, high strain rate, and high temperature. The yield stress is defined accordingly as:

(1) $$\sigma =[A+B\varepsilon_{p}^{n_{p}}][1+C\ln{\dot{\varepsilon }}^{*}][1-T^{*}{}^{m_{t}}]$$

where *A*, *B*, *C*, *n*_p_, and *m*_t_ are experimentally obtained material constants, $\varepsilon_{p}$ is the effective plastic strain, ${\dot{\varepsilon }}^{*}={\dot{\varepsilon }}_{p}/{\dot{\varepsilon }}_{0}$ is the dimensionless equivalent rate when the reference plastic strain rate is ${\dot{\varepsilon }}_{0}=1s^{-1}$, T^*^ = (*T* − *T*_room_)/(*T*_melt_ − *T*_room_) is the dimensionless temperature, and *T*_room_ and *T*_melt_ represent the melting temperature and room temperature, respectively. The detailed parameters for Cu and Al are listed in Table 2.

The explosive 8701 employs the JWL equation of state, which describes the expansion of detonation products for high energy explosives:

(2) $$P=A_{1}(1-\frac{\omega }{R_{1}V})e^{-R_{1}V}+B_{1}(1-\frac{\omega }{R_{2}V})e^{-R_{2}V}+\frac{\omega E_{0}}{V}$$

where *A*_1_, *B*_1_, *R*_1_, *R*_2_, and *ω* are material constants, *E*_0_ represents the detonation energy per unit volume, and V is the relative volume. The material parameters listed in Table 3 [28] are imported into AUTODYN.

### 4.2. Formation, Velocity Gradient, and Velocity Distribution

**Figure 13:**
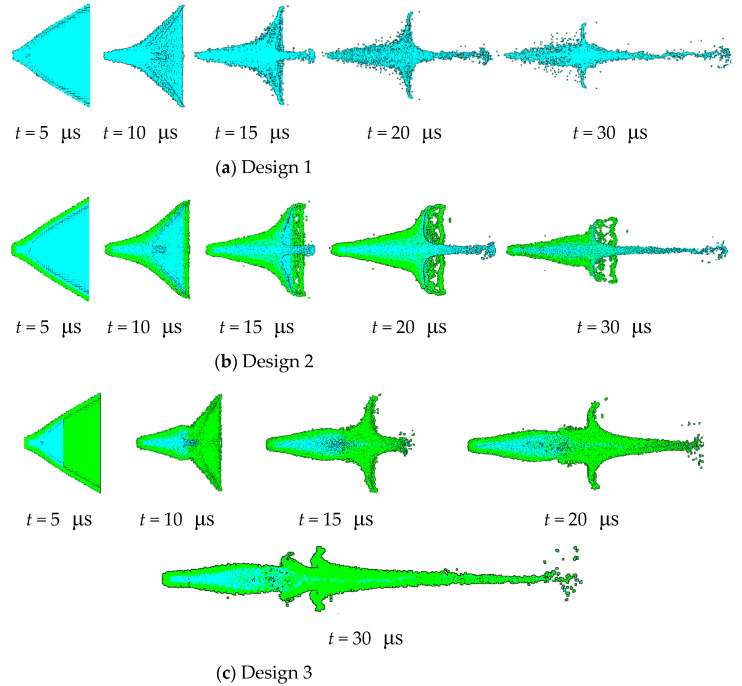
Jet formation by simulations for design 1~3 (**a**–**c**).

Figure 13 illustrates the collapse of the liner and the formation of the jet at typical instants.

The three designs all form high-speed jets from their respective liner. For design 1, the single Cu jet is slender because it is thinner and uses less material, but it easily breaks when the charge is away from the target. In design 2, both the outer Cu layer and the inner Al layer of the liner collapse and deform together. The Cu jet is firstly rapidly extruded to form the tip at *t* = 5 μs and the ensuing Al jet gradually moves to the tail to wrap the Cu tail. In design 3, the outer truncated Cu layer of the liner forms the jet tip under extreme loading, but only the adjacent inner Al layer of the liner gradually covers the Cu jet. At *t* = 10 μs, the jet from the extra inner Al layer that is not attached to Cu at the outset catches up with the jet tip of the original Cu, and a new collision point forms on the axis. As a result, a new jet tip develops at *t* = 15 μs, and the Cu jet begins to recede.

**Figure 14:**
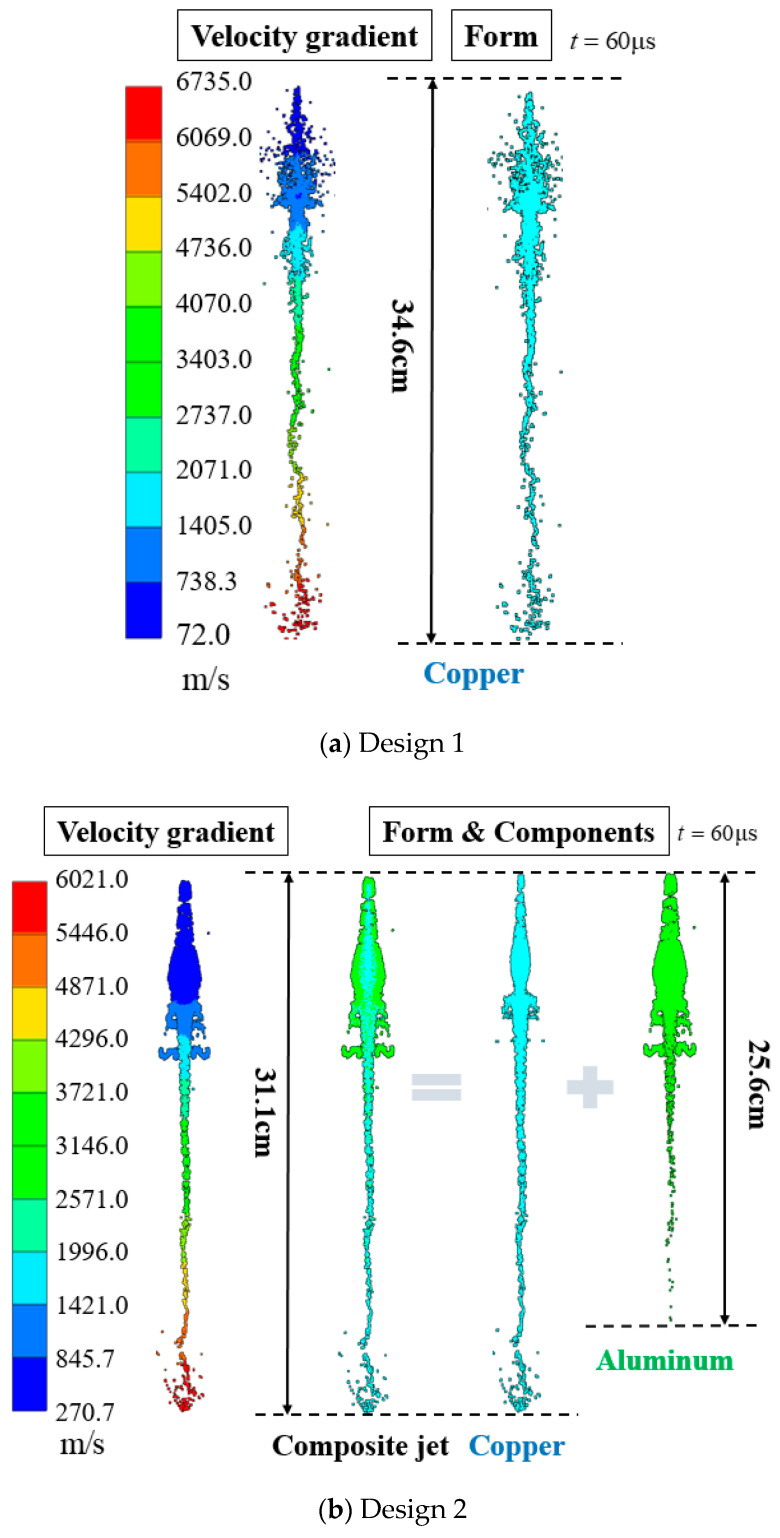

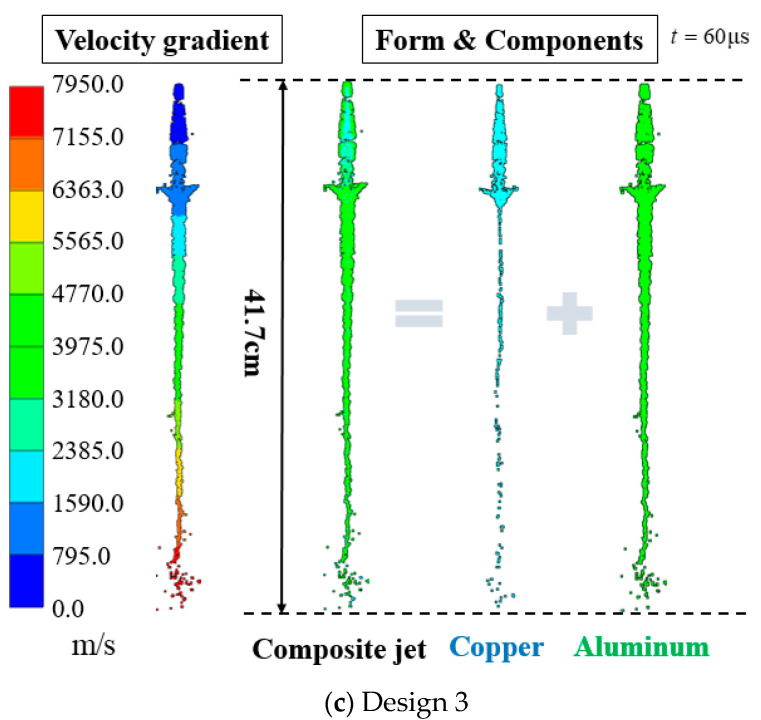
The velocity gradient and distribution of jets in design 1~3 (**a**–**c**).

Figure 14 shows the velocity gradient and the distributions of fully formed jets at *t* = 60 μs for the three designs. The difference between the simulated velocity and the experimentally measured velocity can be ascribed to the impact consumption. For design 1, the jet length is 34.6 cm at *t* = 60 μs, and the tip velocity of the Cu jet reaches 6735 m/s. For design 2, the tip of the composite jet is entirely made of Cu, and the Cu jet is coated with Al in the middle and at the tail. The Cu jet is 31.1 cm in length with a tip velocity of 6021 m/s, whereas the Al jet is only 25.6 cm long, thus demonstrating the substantial difference between the two velocity gradients. In contrast, for design 3, the 41.7 cm long Cu jet is entirely wrapped by Al from the tip to the tail. The velocity gradient is the same for both Cu and Al, with the tip reaching 7950 m/s.

**Figure 15:**
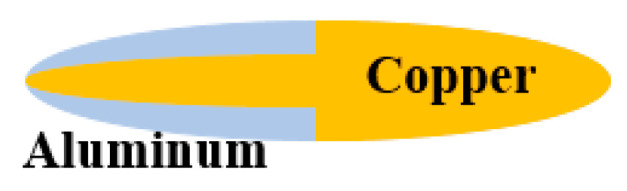
Elemental distribution of composite jet in Design 2.

The different distribution of Cu and Al in the composite jets of design 2 and design 3 should account for the distinct PE effects observed in the experiments. The Cu/Al composite jet in design 3 corresponds to the basic distribution (a) in Section 2. The composite jet in design 2 is a combination of (a) and (c), which can be illustrated in a more straightforward way in Figure 15. Their different PE effects are well demonstrated by the penetration experiments, from the increasing amplitude of perforation and deformation as well as the profile and synchronization of firelight recorded by high-speed photography.

## 5. Conclusions

This paper provided penetration experiments for a single Cu jet and two composite jets composed of Cu and Al using multilayer targets to investigate PE effects. The following conclusion can be made.

1. Significant PE effects were observed for the Cu/Al composite jets according to the perforation and deformation of the target plates and the observed firelight characteristics.
2. The two composite jets are distinct in their effects, including perforation, deformation, and synchronous firelight. The distinct PE effects can be attributed to the different elemental distributions. Simulation shows that for design 2, the tip of the composite jet is exclusively Cu and the rest of the jet is wrapped with Al. In contrast, for design 3, the Cu jet is almost wrapped entirely in Al from the tip to the tail.
3. The PE effects of four basic distributions in composite jets are analyzed according to different damaging characteristics of the inactive and active parts. The Cu/Al composite jet in design 3 corresponds to the basic distribution (a), whereas design 2 corresponds to a combination of (a) and (c). Design 2 differs from design 3 only from its full-height Cu liner, and consequently cause different elemental distributions of the jets, which leads to poorer penetration depth and lateral damage.

It can thus be seen that simply increasing the active component can not necessarily improve the aftereffect, since efficient combination of the inactive and active components in well-designed double-layer liner is indispensable for good PE effects. This paper can provide a useful tool for optimizing the aftereffect enhancement of RSMs applied in shaped charge.

## Figures and Tables

**Table 1:** Experimental conditions.

No.	Main Charge		Inner Liner				Outer Liner			
	Material	Mass/g	Material	Height/mm	Width/mm	Mass/g	Material	Height/mm	Width/mm	Mass/g
Design 1	JH-2	175.14	/				Cu	42.0	54.2	41.1
Design 2		175.16	Al	42.0	54.2	25.1		40.0	49.5	32.8
Design 3		175.12				25.9		20.0	26.4	9.2

**Table 2:** Parameters of Cu and Al.

Materials	ρ /(g·cm^−3^)	*A*/GPa	*B*/GPa	*C*	*n*_p_	*m*_t_
Copper	8.96	0.090	0.292	0.025	0.31	1.09
Al	2.77	0.337	0.343	0.01	0.41	1.00

**Table 3:** Parameters of explosive 8701 [28].

Material	ρ /(g·cm^−3^)	*A*/GPa	*B*/GPa	*R*_1_	*R*_2_	ω	*E*_0_/GPa	*P*_CJ_/GPa	*D*/(m·s^−1^)
explosive 8701	1.71	524.23	7.678	4.2	1.1	0.34	8.499	28.6	8315

## Data Availability

Not applicable.
